# A simple overflow density separation method that recovers >95% of dense microplastics from sediment

**DOI:** 10.1016/j.mex.2024.102638

**Published:** 2024-02-27

**Authors:** Thomas W. Crutchett, Katrina R. Bornt

**Affiliations:** School of Biological Sciences and the Oceans Institute, The University of Western Australia, 35 Stirling Highway, Crawley, Western Australia 6009, Australia

**Keywords:** Overflow density separation method, Plastic pollution, Protocol validation, Microplastic extraction, Environmental contamination, Zinc chloride

## Abstract

Density separation can isolate microplastics from environmental samples containing sediment. Typically, a solution added to sediment causes microplastics with lower densities to float. The solution of choice can influence the recovery of different particles since denser solutions can separate a greater range of microplastics. The equipment and procedural complexity further influence density separation protocols and microplastic recoveries. Zinc chloride (ZnCl_2_) is frequently used to isolate high-density polymers from environmental samples yet is rarely validated with simple, well-described protocols. A simple overflow method using ZnCl_2_ to isolate microplastics from sediment samples is described following a 3-step process: (1. Separation) ZnCl_2_ (1.7 g cm^−3^) solution is added to a sediment sample, agitated then settled; (2. Overflows) buoyant particles at the surface of the solution are overflowed twice; (3. Filtration) the overflowed solution is filtered. In a validation experiment with polyamide, rubber, polyvinyl chloride and polyethylene terephthalate/polyester, the mean recovery using this overflow method was 96 % ± 0.6 (standard error). This overflow density separation method proposes an accessible and reliable protocol to extract medium and high-density microplastics.

• Microplastic separation with concentrated ZnCl_2_ solution
• Simple overflow of buoyant particles
• Reliable extraction of microplastics

## Graphical abstract

**Figure:**
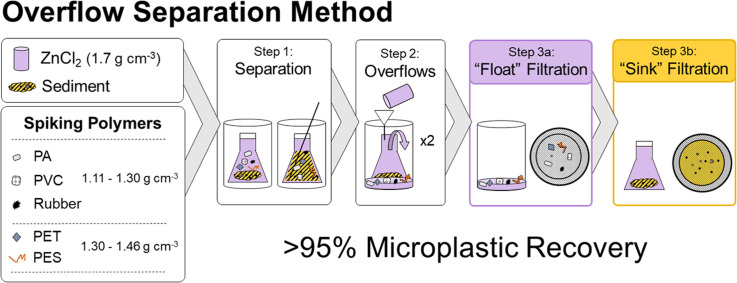

Specifications Table

**Table:** 

Subject area:	Environmental Science
More specific subject area:	Microplastic extraction
Name of your method:	Overflow density separation method
Name and reference of original method:	This method describes a simple protocol that efficiently extracts high-density microplastics from sediment. It is a modification of the ZnCl_2_ density separation presented in: T. Mani, S. Primpke, C. Lorenz, G. Gerdts, P. Burkhardt-Holm, Microplastic Pollution in Benthic Midstream Sediments of the Rhine River, Environ. Sci. Technol. 53 (2019) 6053–6062.
Resource availability:	*Specific resources* ZnCl_2_ solution (1.7 g cm^−3^*)* 500 ml polyethylene laboratory wash bottles (ZnCl_2_ and H_2_O solutions) 400 ml glass beaker (tall form, Ø 70 mm; H 130 mm) 50 ml glass Erlenmeyer flask (narrow neck, upper Ø 22 mm) Glass funnel (upper Ø 60 mm, lower Ø 5 mm) Ø 25 mm & 47 mm glass vacuum filtration systems (Merck Millipore) Stainless steel mesh filters (Sefar Pty Ltd, twill weave, Ø 25 mm, 25 µm pore size) Glass fibre filters (MN GF-4; Ø 47 mm, 1.4 µm pore size) Glass Petri dishes (Ø 40 mm and Ø 60 mm)

## Method details

### Overview

Density separation is routinely used to assist in the extraction of microplastics from environmental samples with inorganic material such as sediment [1]. Microplastics that are negatively buoyant (i.e. sink) in tap water (e.g. polyamide, PA; polyethylene terephthalate, PET; polycarbonate, PC; polyvinyl chloride, PVC) can be separated from sediment when mixed with a salt solution of a higher density. Saturated sodium chloride (NaCl; 1.2 g cm^−3^) is commonly used to extract microplastics, however, other salt solutions (e.g., zinc chloride; ZnCl_2_, sodium iodide; NaI) are effective at suspending a greater range of polymer types, including those with higher densities (e.g. PVC, PET) [2,3] and less impacts on polymers [4]. The type of salt solution, therefore, influences microplastic recoveries and contributes to the lack of harmonisation among different density separation methods [5,6]. Additionally, other cost, equipment availability, and environmental and safety factors must be considered when selecting the appropriate salt solution.

Many density separation methods stem from a relatively simple process described by Thompson et al. [7], where sediment samples are mixed with concentrated saline solutions and allowed to settle before the supernatant fraction is decanted and filtered. However, decanting the supernatant fraction can cause plastic particles to adhere to the apparatus during pouring and therefore, reduce the number of microplastics extracted [1]. Microplastic recovery is improved by repeating the agitation, settling, and decant process, but this requires additional processing time and can increase the risk of particle loss [8], [9], [10]. Separatory funnels can be used to isolate the supernatant by draining the settled material from the bottom of the vessel [11], [12], [13]; however, this often causes blockages [3]. A range of specialised apparatus and procedures have been developed to extract plastic from sediment and navigate the challenges associated with efficiency, particle loss and blockages [8,^14^,15]. Unfortunately, specialised equipment is often expensive and sometimes requires complex operating procedures [6,16].

Alternatively, microplastics can be separated from sediment by overflowing a plastic-containing supernatant into a separate collection vessel [17]. Overflow separation offers many benefits given it requires only standard laboratory equipment and can be adjusted to suit research objectives and limitations (e.g. sample mass, polymers of interest, chemical and equipment availability). Overflow separation methods are effective at isolating microplastics from sediment samples [17], [18], [19], [20], [21], [22], yet detailed procedures are limited and rarely validated with a range of high-density polymers. In addition, overflow separation methods seldom use solutions prepared at high densities that provide a greater recovery of microplastics [2]. Here, we present an overflow separation method, adapted from existing methods [17], that uses common laboratory equipment and ZnCl_2_ (1.7 g cm^−3^) to isolate medium and high-density microplastics from sediment.

## Setup

Details of the contamination controls and sediment preparation used for this procedure validation are provided in the Supplementary Material.

## Procedure

*Step 1 - Separation*

1. Add approximately 10 g of sediment to a 50 ml Erlenmeyer flask (Fig. 1a).

**Fig. 1:**
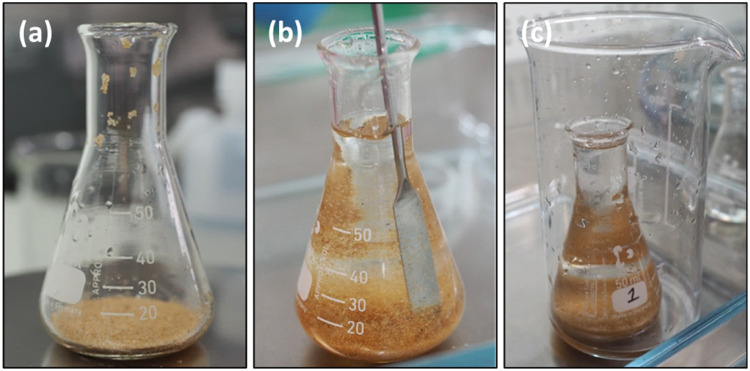
Sediment in an Erlenmeyer flask (a), with agitation in a ZnCl_2_ solution (b), and position in an overflow beaker (c) during Step 1 of the overflow density separation method.
2. Use ≤ 25 ml of ZnCl_2_ from the wash bottle to carefully rinse the inside of the flask and wash down any adhering particles.
3. Thoroughly agitate the sample using a spatula (or similar) for one minute. It is important to gently stir the solution in a non-uniform motion to prevent the formation of a vortex, which interferes with particle suspension (Fig. 1b). Once agitated, carefully remove the spatula and rinse it with a small amount of ZnCl_2_ to return any adhering particles to the Erlenmeyer flask.
4. Carefully lift and rinse the outside of the Erlenmeyer flask with filtered water before placing it inside a 400 ml glass beaker.
5. Fill the Erlenmeyer flask with additional ZnCl_2_ to the base of the neck whilst disturbing any particles caught in the surface tension of the solution.
6. Complete another agitation with the spatula as described above. Be mindful not to rinse the spatula too much and overflow the solution early. If too much ZnCl_2_ is already in the flask, rinse the spatula into the overflow beaker instead.
7. Add ZnCl_2_ to 1 cm below the top of the Erlenmeyer flask, if required (Fig. 1c). It may be necessary to add a few drops of ZnCl_2_ to help disperse any surface sediment particles present shortly after agitation. Allow at least 20 min for the particles to separate before proceeding with the overflows. The appropriate wait time between overflows depends on solution clarity and operational preferences.

*Step 2 - Overflows*

8. *First overflow*: Ensure the bottom of the Erlenmeyer flask is touching one wall of the beaker to encourage floating particles to flow over one edge whilst limiting the area for particles to adhere. Submerge the tip of the funnel approximately 1 cm into the supernatant. Position the funnel tip away from the edge that will overflow into the beaker and gently pour 15 ml of ZnCl_2_ at approximately 1 ml/second (Fig. 2a). If the ZnCl_2_ is poured too quickly or the funnel is positioned too low, settled material may become suspended, and the process will need to be paused until the sediment resettles.

**Fig. 2:**
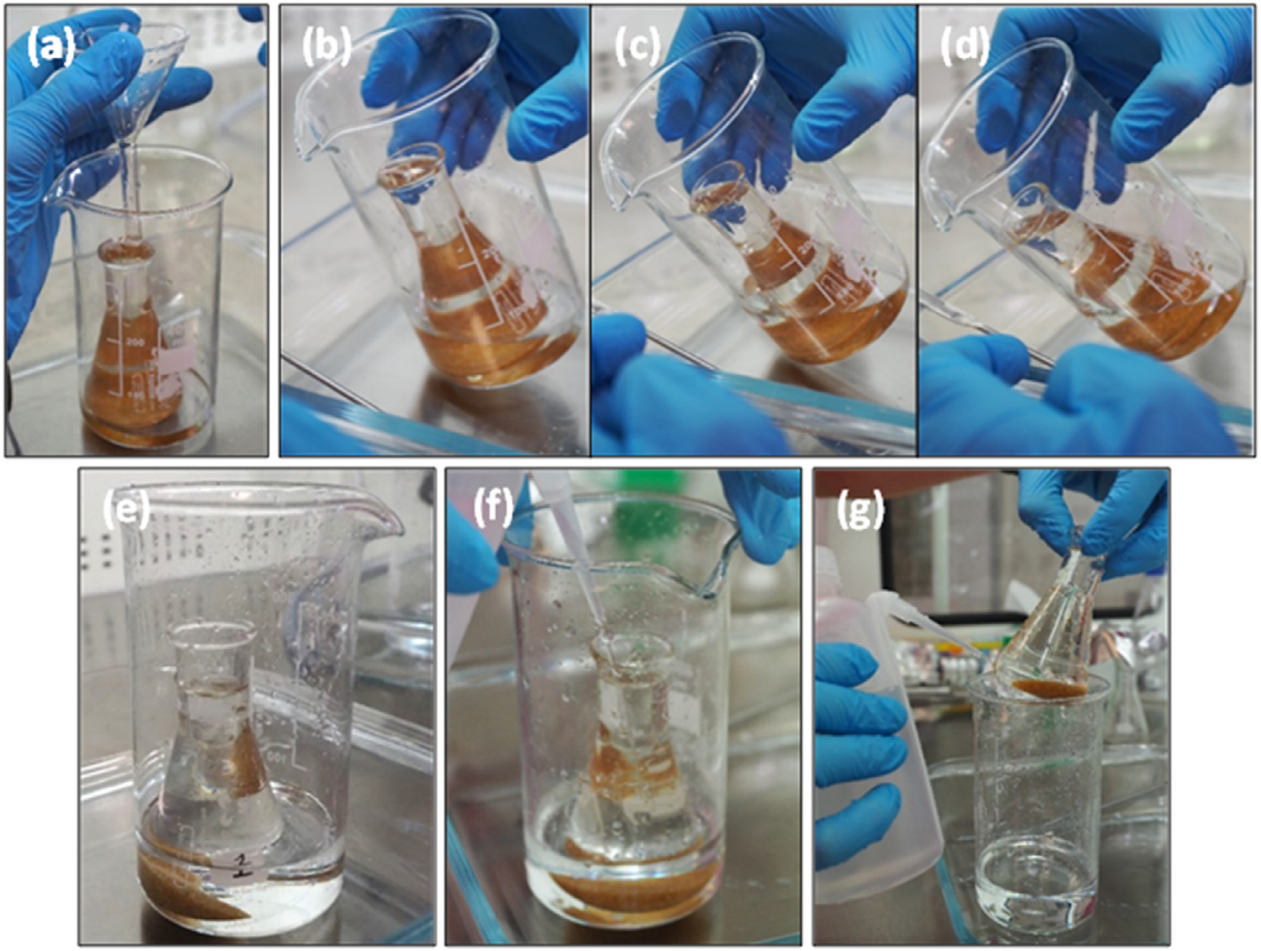
The position of the funnel tip (a), tilting of the overflow beaker (b-d), resulting separation (e), and rinsing the top (f) and bottom (g) of the Erlenmeyer flask during Step 2 of the overflow density separation method.
9. Remove the end of the funnel from the solution and rinse it with ZnCl_2_ to wash any adhering particles into the overflow beaker.
10. Gently tilt the beaker until the top edge of the Erlenmeyer flask touches the inside wall of the beaker, then allow approximately 2 cm of the supernatant to overflow into the beaker (Fig. 2b-d). If settled particles are disturbed when tilting, stop tilting the flask and let the contents resettle before reattempting. Removing additional supernatant allows the sample to be agitated again before the second overflow.
11. *Second overflow*: conduct another agitation and allow the solution to settle (as above) before collecting a second overflow (Fig. 2e).
12. Rinse the outside top half of the Erlenmeyer flask with ZnCl_2_ whilst it remains in the beaker, being careful not to fill the flask and cause another overflow (Fig. 2f). The rinsing of the Erlenmeyer flask is critical to ensure no particles are lost. Be sure to rinse the top edge to remove any particles adhering to the rim of the flask.
13. Raise the Erlenmeyer flask out of the overflow solution and rinse the bottom outside half of the glass into the overflow beaker with ZnCl_2_ (Fig. 2g). Special attention should be taken when rinsing the underside of the flask. The overflow beaker now contains the “float” fraction to be filtered for microplastic recovery (Step 3a).
14. Set aside the Erlenmeyer flask that now contains the “sink” fraction (Step 3b).

*Step 3a - Float Filtration*

15. Whilst under vacuum, pour the contents from the overflow beaker onto the centre of the filter (Ø 25 mm). Pour slowly (∼ 1 ml/second) to avoid any solution building up on the filter (Fig. 3a).

**Fig. 3:**
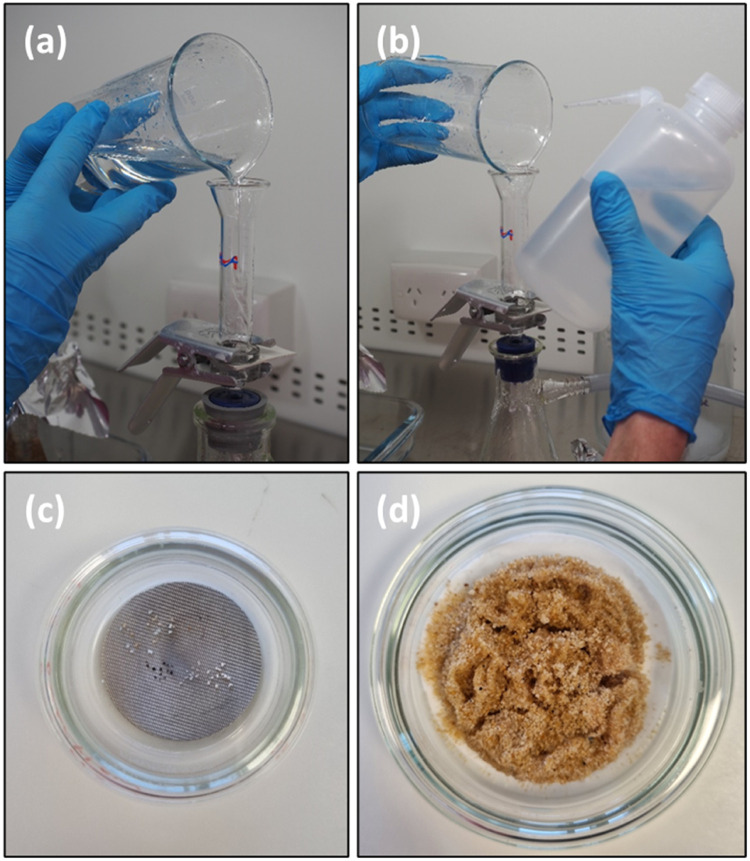
Filtering (a) and rinsing (b) the overflow beaker (Step 3a) onto a Ø 25 mm filter to isolate particles (c). The sink fraction (Step 3b) is then filtered onto a Ø 47 mm filter (d).
16. Thoroughly rinse the inside of the overflow beaker with filtered H_2_O to wash all particles from the “float” fraction into the filtration funnel. Rinse the filtration funnel with additional filtered H_2_O to wash adhered particles onto the filter and remove residual ZnCl_2_ (Fig. 3b).
17. Turn the vacuum off and carefully remove the filtration funnel. Check for any visible particles that may be adhered to the inner edge of the funnel base. Remove any adhered particles by gently brushing them onto the filter using a fine-tipped probe. Carefully remove the filter with fine tweezers and place it in a glass Petri dish (Fig. 3c).
18. Inspect the filtration funnel again using a stereo-microscope and immediately relocate any particles onto the filter to avoid airborne contamination.
19. Dry the glass Petri dish containing the filter in an oven set to ≤ 50 °C for 24 h with the lid offset and covered in aluminium foil, or enclose it in a larger glass Petri dish.

*Step 3b - Sink Filtration*

20. Vacuum filter the Erlenmeyer flask containing the “sink” fraction onto a glass fibre filter (Ø 47 mm).
21. Rinse any remaining sediment into the vacuum filtration funnel with filtered H_2_O. Once all of the sediment is on the filter, thoroughly rinse it with filtered H_2_O to remove any residual ZnCl_2_. Remove the filter from the vacuum setup and transfer it to a glass Petri dish (Fig. 3d). Ensure all the sink material is collected and dry it in an oven as described in Step 3a.

## Procedure validation

The efficacy of removing microplastics with this overflow density separation method was validated by spiking and then recovering medium and high-density microplastics from sediment. Five replicate density separations were conducted using sediment spiked with visually distinctive microplastics. PA, crumbed rubber (CR), PVC, PET and polyester (PES) 500 µm < 1000 µm in length were used to determine the percent recovery of plastic with increasing densities (Table 1). Polymer densities can broadly vary from manufacturing additives, weathering and biofouling [1]; therefore, density ranges were identified for the spiking polymers (Table 1) by testing their buoyancy in incremental ZnCl_2_ concentrations (1.00 - 1.60 g cm^−3^). The number of spiked polymers were counted and verified by two analysts who independently inspected each float filter (Fig. 3c) [6].

**Table 1:** Reference polymers used for spiking to validate the overflow density separation method. Orange scale bars on reference images represent 1 mm.

## Validation results

**Fig. 4:**
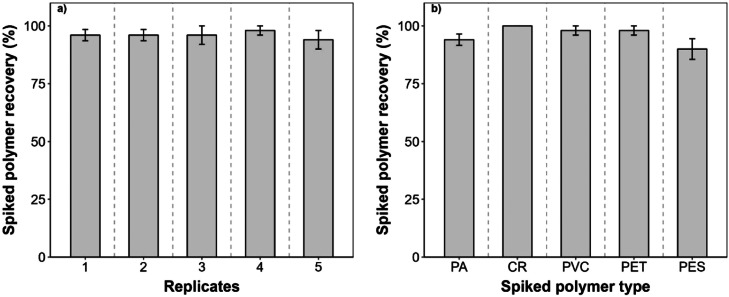
The mean (± standard error) percentage of spiked polymers recovered from each replicate sample (a) and for each polymer type (b). PA, polyamide; CR, crumbed rubber; PVC, polyvinyl chloride; PET, polyethylene terephthalate; PES, polyester.

On average, 96 % ± 0.6 (standard error; SE) of all spiked polymers were recovered from each replicate sample (Fig. 4a). There was no significant difference between the total recovery of all spiked polymers across replicates (ANOVA, stat 0.04, *p*-value 1.00). The mean recoveries of PA (94 % ± 0.49 SE), CR (100 % ± 0 SE), PVC (98 % ± 0.40 SE), PET (98 % ± 0.40 SE), PES (90 % ± 0.89 SE) (Fig. 4b) were not significantly different (ANOVA, stat 0.34, *p*-value 0.99).

## Additional considerations

### Preliminary assessment

Plastic contamination in sediment is potentially underestimated in studies that apply methods that are unable to extract high-density plastics [1,6]. Using basic laboratory equipment, we developed an overflow density separation method to isolate microplastics from sediment. A preliminary trial of the overflow method was conducted to compare the efficiency of NaCl and ZnCl_2_ solutions. NaCl was excluded from further considerations after failing to suspend PVC, PET, and PES (Table S1). The procedure validation was therefore, conducted with ZnCl_2_, a solution commonly recommended for density separation [4], which can also be recycled to reduce waste production and costs [23].

### Method application and development

We recommend that future users of this method conduct preliminary validation tests to determine whether it is suitable or requires any procedural adjustments. For example, samples with high organic matter may require digestion before density separation [24]. Organic matter was mostly absent from the trial sand used for validating this method, and therefore, it did not require pre-treatment (e.g. chemical digestion). It is recognised that this procedure, developed with 10 g sediment samples (or subsamples), can be upscaled and modified to accommodate glassware availabilities, sample sizes or treatment preferences but would require further validation. There are also opportunities to explore the recovery of additional size classes and the functionality of this procedure for coarser or finer sediment and other sample matrices (e.g. to isolate microplastics from ingested inorganic material). While operational optimisation was not the focus of this study, similar process times have been reported [8]. Further refinement of the process (e.g. density separating in batches) would likely reduce the time requirements and improve overall efficiency when preparing samples for microplastics analysis.

### Sample analysis

Isolating suspected microplastic particles from sediment is the general purpose of density separation. By correctly following the overflow density separation method presented here, two fractions are generated (i.e. float and sink fraction). The float fraction, filtered on a Ø 25 mm stainless steel filter (Fig. 3c), contains limited inorganic material and provides a well clarified filter matrix that makes it compatible with spectroscopic techniques used to identify microplastics. Further using a smaller diameter filter (Ø 25 mm relative to Ø 47 mm) concentrates microplastics over a smaller surface area, therefore, facilitating their extraction for Fourier Transform Infrared (FTIR) Spectroscopy. Both the small diameter of filters and the use of stainless steel are also favourable for Raman Spectroscopy. The sink fraction, filtered on a larger (Ø 47 mm) glass fibre filter (Fig. 3d), will contain the bulk of the sediment sample (e.g. sand, silt, clay) and should not require spectroscopic analysis but can be retained, if necessary. The difference between the clarity of filters from the float and sink fraction (e.g. Fig. 3c and d) is very apparent and highlights the effectiveness of this overflow density separation method at isolating microplastics from the majority of inorganic material.

## Ethics statements

N/A

## Funding

This research was funded by the Jock Clough Marine Foundation, through the Robson & Robertson Young Scholar Program and the Holsworth Wildlife Research Endowment – Equity Trustees Charitable Foundation & the Ecological Society of Australia. The Department of Biodiversity, Conservation and Attractions also supported this research through a funding agreement with the Rivers and Estuaries Science program. The authors were recipients of a University Postgraduate Award at The University of Western Australia with T. Crutchett additionally supported by the Department of Water and Environmental Regulation.

## CRediT authorship contribution statement

**Thomas W. Crutchett:** Conceptualization, Methodology, Software, Validation, Formal analysis, Investigation, Resources, Data curation, Writing – original draft, Writing – review & editing, Visualization, Project administration, Funding acquisition. **Katrina R. Bornt:** Conceptualization, Methodology, Software, Validation, Formal analysis, Investigation, Resources, Data curation, Writing – original draft, Writing – review & editing, Visualization, Project administration, Funding acquisition.

## Declaration of competing interest

The authors declare that they have no known competing financial interests or personal relationships that could have appeared to influence the work reported in this paper.

## Data Availability

Data will be made available on request.

## Appendix Supplementary materials

Image, application 1

## References

[1] Hidalgo-Ruz V., Gutow L., Thompson R.C., Thiel M. (2012). Microplastics in the marine environment: a review of the methods used for identification and quantification. Environ. Sci. Technol..

[2] Quinn B., Murphy F., Ewins C. (2017). Validation of density separation for the rapid recovery of microplastics from sediment. Anal. Methods.

[3] Nuelle M.T., Dekiff J.H., Remy D., Fries E. (2014). A new analytical approach for monitoring microplastics in marine sediments. Environ. Pollut..

[4] Schrank I., Möller J.N., Imhof H.K., Hauenstein O., Zielke F., Agarwal S., Löder M.G.J., Greiner A., Laforsch C. (2022). Microplastic sample purification methods - Assessing detrimental effects of purification procedures on specific plastic types. Sci. Total Environ..

[5] Cashman M.A., Ho K.T., Boving T.B., Russo S., Robinson S., Burgess R.M. (2020). Comparison of microplastic isolation and extraction procedures from marine sediments. Mar. Pollut. Bull..

[6] Way C., Hudson M.D., Williams I.D., Langley G.J. (2022). Evidence of underestimation in microplastic research: a meta-analysis of recovery rate studies. Sci. Total Environ..

[7] Thompson R.C., Olsen Y., Mitchell R.P., Davis A., Rowland S.J., John Anthony W.G., McGonigle D., Russell A.E. (2004). Lost at sea: where is all the plastic?. Science.

[8] Enders K., Lenz R., Ivar do Sul J.A., Tagg A.S., Labrenz M. (2020). When every particle matters: a QuEChERS approach to extract microplastics from environmental samples. MethodsX.

[9] Dimante-Deimantovica I., Suhareva N., Barone M., Putna-Nimane I., Aigars J. (2022). Hide-and-seek: threshold values and contribution towards better understanding of recovery rate in microplastic research. MethodsX.

[10] Besley A., Vijver M.G., Behrens P., Bosker T. (2017). A standardized method for sampling and extraction methods for quantifying microplastics in beach sand. Mar. Pollut. Bull..

[11] Masura J., Baker J., Foster G., Arthur C. (2015).

[12] Fries E., Dekiff J.H., Willmeyer J., Nuelle M.T., Ebert M., Remy D. (2013). Identification of polymer types and additives in marine microplastic particles using pyrolysis-GC/MS and scanning electron microscopy. Environ. Sci. Process. Impacts.

[13] Wazne M., Mermillod-Blondin F., Vallier M., Krause S., Barthélémy N., Simon L. (2024). Optimization of glass separating funnels to facilitate microplastic extraction from sediments. MethodsX.

[14] Claessens M., Van Cauwenberghe L., Vandegehuchte M.B., Janssen C.R. (2013). New techniques for the detection of microplastics in sediments and field collected organisms. Mar. Pollut. Bull..

[15] Imhof H.K., Schmid J., Niessner R., Ivleva N.P., Laforsch C. (2012). A novel, highly efficient method for the separation and quantification of plastic particles in sediments of aquatic environments. Limnol. Oceanogr. Methods.

[16] Coppock R.L., Cole M., Lindeque P.K., Queirós A.M., Galloway T.S. (2017). A small-scale, portable method for extracting microplastics from marine sediments. Environ. Pollut..

[17] Mani T., Primpke S., Lorenz C., Gerdts G., Burkhardt-Holm P. (2019). Microplastic pollution in benthic midstream sediments of the Rhine river. Environ. Sci. Technol..

[18] Sánchez-Nieva J., Perales J.A., González-Leal J.M., Rojo-Nieto E. (2017). A new analytical technique for the extraction and quantification of microplastics in marine sediments focused on easy implementation and repeatability. Anal. Methods.

[19] Strady E., Dang T.H., Dao T.D., Dinh H.N., Do T.T.D., Duong T.N., Duong T.T., Hoang D.A., Kieu-Le T.C., Le T.P.Q., Mai H., Trinh D.M., Nguyen Q.H., Tran-Nguyen Q.A., Tran Q.V., Truong T.N.S., Chu V.H., Vo V.C. (2021). Baseline assessment of microplastic concentrations in marine and freshwater environments of a developing Southeast Asian country, Viet Nam. Mar. Pollut. Bull..

[20] Rivoira L., Castiglioni M., Rodrigues S.M., Freitas V., Bruzzoniti M.C., Ramos S., Almeida C.M.R. (2020). Microplastic in marine environment: reworking and optimisation of two analytical protocols for the extraction of microplastics from sediments and oysters. MethodsX.

[21] Vermeiren P., Muñoz C., Ikejima K. (2020). Microplastic identification and quantification from organic rich sediments: a validated laboratory protocol. Environ. Pollut..

[22] Horton A.A., Svendsen C., Williams R.J., Spurgeon D.J., Lahive E. (2017). Large microplastic particles in sediments of tributaries of the River Thames, UK - Abundance, sources and methods for effective quantification. Mar. Pollut. Bull..

[23] Rodrigues M.O., Gonçalves A.M.M., Gonçalves F.J.M., Abrantes N. (2020). Improving cost-efficiency for MPs density separation by zinc chloride reuse. MethodsX.

[24] Prata J.C., da Costa J.P., Duarte A.C., Rocha-Santos T. (2019). Methods for sampling and detection of microplastics in water and sediment: a critical review. Trends Analyt. Chem..

