# Supplementary material for: A simple overflow density separation method that recovers >95% of dense microplastics from sediment
Source: MethodsX. 2024 Feb 27;12:102638. doi: 10.1016/j.mex.2024.102638 (PMC10912609; doi:10.1016/j.mex.2024.102638)
Supplement: Supplementary file 1 [file mmc1.docx]

**Supplementary Material**

A simple overflow density separation method that recovers >95% of dense microplastics from sediment

Thomas W Crutchett ^a^, *Katrina R Bornt ^a^

^a^ School of Biological Sciences and the Oceans Institute, The University of Western Australia, 35 Stirling Highway, Crawley, Western Australia 6009, Australia

* Corresponding author: tel: + 618 6488 6000; e-mail: katrina.bornt@research.uwa.edu.au.

**Contamination mitigation**

We applied strict measures in this procedure to reduce microplastic contamination from external sources. The process was conducted in a restricted-access laboratory where researchers routinely wipe all surfaces and use a HEPA-filtered stick vacuum (Samsung™). Researchers wore 100% cotton pink (dyed) lab coats and nitrile (powder-free) gloves. All work was conducted in a laminar flow cabinet (laftech) where possible, with glass lids or aluminium foil used to prevent airborne contamination from entering vessels. All working and rinse solutions were prefiltered through 1.4 µm pore size MACHEREY-NAGEL glass fibre filters (MN GF-4). Glassware and utensils were thoroughly washed using detergent before being triplicate rinsed with filtered Deionised (DI) water and air dried in a laminar flow cabinet. Stainless steel filters were pre-rinsed with filtered DI and inspected using a stereo-microscope immediately before use. Two procedural blanks were incorporated in the validation trials to monitor potential contamination and eliminate it from the sample replicates.

**Trial sand preparation**

Approximately 0.5 kg of sand was collected from an estuarine beach of the Swan River estuary (31°58'49.0"S 115°49'15.7"E) for use in the validation trials. The sand was taken from an area above the tideline by collecting multiple grab samples from the top 2 cm surface layer. Sand with minimal organic debris was collected to eliminate pre-treatment chemical digestion requirements for samples. The sand was dried at 50°C before being hand-screened in 100 g portions using 0.106-, 0.250-, 0.500-, and 1.000-mm stainless steel sieves. Any material that was <0.106 mm or >1.000 mm was discarded. The material retained on the 0.106-, 0.250-, and 0.500-mm sieves was thoroughly homogenised in a stainless-steel bowl. 10 g subsamples of the homogenised trial sand were used for all separations, excluding the procedural blank.

**Spiking polymers**

Visually distinctive spiking plastics and chemical confirmation ensured particle counts were accurate and not influenced by plastics present in the trial sand. The chemical composition of the spiking polymers were confirmed by fourier transform infrared spectroscopy with a Thermo Scientific™ Nicolet™ iS50 from attenuated total reflectance (ATR) (16 scans at 4 cm-1 resolution, wave number range = 4000 - 400 cm-1) against known reference polymers [1].

| **Table S1.** Recovery of polymers spiked in NaCl and ZnCl_2_ solutions using the overflow density separation method. PA, polyamide; CR, crumbed rubber; PVC, polyvinyl chloride; PET, polyethylene terephthalate; PES, polyester. | | |
| --- | --- | --- |
|  | Salt solutions | |
| Spiking polymer types | NaCl (1.2 g cm^-3^) | ZnCl_2_ (1.7 g cm^-3^) |
| PA | 10 | 10 |
| CR | 7 | 10 |
| PVC | 0 | 10 |
| PET | 0 | 10 |
| PES | 0 | 8 |
| total recovery | 17 | 48 |
| % recovery | 34 | 94 |

**References**

[1] Center for Marine Debris Research, Polymer Kit 1.0 to Harmonize Plastic Pollution Research, (2021). https://www.hpu.edu/cncs/cmdr/img/polymerkit1.0_marketingbrochure.pdf (accessed October 2023).
